# Characterizing compatibility and agreement of unrooted trees via cuts in graphs

**DOI:** 10.1186/1748-7188-9-13

**Published:** 2014-04-17

**Authors:** Sudheer Vakati, David Fernández-Baca

**Affiliations:** 1 Department of Computer Science, Iowa State University, Ames, Iowa, USA

**Keywords:** Phylogenies, Supertrees, Compatibility, Agreement, Cuts in graphs, Chordal graphs

## Abstract

**Background:**

Deciding whether there is a single tree —a supertree— that summarizes the evolutionary information in a collection of unrooted trees is a fundamental problem in phylogenetics. We consider two versions of this question: agreement and compatibility. In the first, the supertree is required to reflect precisely the relationships among the species exhibited by the input trees. In the second, the supertree can be more refined than the input trees.

Testing for compatibility is an NP-complete problem; however, the problem is solvable in polynomial time when the number of input trees is fixed. Testing for agreement is also NP-complete, but it is not known whether it is fixed-parameter tractable. Compatibility can be characterized in terms of the existence of a specific kind of triangulation in a structure known as the display graph. Alternatively, it can be characterized as a chordal graph sandwich problem in a structure known as the edge label intersection graph. No characterization of agreement was known.

**Results:**

We present a simple and natural characterization of compatibility in terms of minimal cuts in the display graph, which is closely related to compatibility of splits. We then derive a characterization for agreement.

**Conclusions:**

Explicit characterizations of tree compatibility and agreement are essential to finding practical algorithms for these problems. The simplicity of the characterizations presented here could help to achieve this goal.

## Background

A *phylogenetic treeT* is an unrooted tree whose leaves are bijectively mapped to a label set ℒ(T). Labels represent species and *T* represents the evolutionary history of these species. Let  be a collection of phylogenetic trees. We call  a *profile*, refer to the trees in  as *input trees*, and denote the combined label set of the input trees, $\underset{T\in P}{⋃}ℒ(T)$, by ℒ(P). A *supertree* of  is a phylogenetic tree whose label set is ℒ(P). The goal of constructing a supertree for a profile is to synthesize the information in the input trees in a larger, more comprehensive, phylogeny [1]. Ideally, a supertree should faithfully reflect the relationships among the species implied by the input trees. In reality, this is rarely achievable, because of conflicts among the input trees due to errors in constructing them or to biological processes such as lateral gene transfer and gene duplication.

We consider two classic versions of the supertree problem, based on the closely related notions of compatibility and agreement. Let *S* and *T* be two phylogenetic trees where ℒ(T)⊆ℒ(S) —for our purposes, *T* would be an input tree and *S* a supertree. Let *S*^′^ be the tree obtained by suppressing any degree-two vertices in the minimal subtree of *S* connecting the labels in ℒ(T). We say that *SdisplaysT*, or that *T* and *S* are *compatible*, if *T* can be derived from *S*^′^ by contracting edges. We say that tree *T* is an *induced subtree* of *S*, or that *T* and *Sagree*, if *S*^′^ is isomorphic to *T*.

Let  be a profile. The *tree compatibility problem* asks if there exists a supertree for  that displays all the trees in . If such a supertree *S* exists, we say that  is *compatible* and *S* is a *compatible supertree* for . The *agreement supertree problem* asks if there exists a supertree for  that agrees with all the trees in . If such a supertree *S* exists, we say that *S* is an *agreement supertree* (AST) for .

Compatibility and agreement embody different philosophies about conflict. An agreement supertree must reflect precisely the evolutionary relationships exhibited by the input trees. In contrast, a compatible supertree is allowed to exhibit more fine-grained relationships among certain labels than those exhibited by an input tree. From a biological viewpoint, the differences between compatibility and agreement reflect different ways to treat *polytomies* —i.e., nodes of degree greater than three. Compatibility treats polytomies as *soft* facts: if an input tree node has degree four or more, it is not because there were multiple simultaneous speciation events, but because there is not enough information to resolve the sequence of speciation. Thus, if another input tree provides more refined information about speciation order, we can use it, provided the information is not contradicted by the remaining input trees. Agreement, in contrast, treats polytomies as *hard* facts. Note that compatibility and agreement are equivalent when the input trees are binary.

If all the input trees share a common label (which can be viewed as a root node), both tree compatibility and agreement are solvable in polynomial time [2,3]. In general, however, the two problems are NP-complete, and remain so even when the trees are quartets; i.e., binary trees with exactly four leaves [4]. Nevertheless, Bryant and Lagergren showed that the tree compatibility problem is fixed-parameter tractable when parametrized by number of trees [5]. It is unknown whether or not the agreement supertree problem has the same property.

To prove the fixed-parameter tractability of tree compatibility, Bryant and Lagergren first showed that a necessary (but not sufficient) condition for a profile to be compatible is that the tree-width of a certain graph —the *display graph* of the profile (see Section ‘Display graphs and edge label intersection graphs’)— be bounded by the number of trees. They then showed how to express compatibility as a bounded-size monadic second-order formula on the display graph. By Courcelle’s Theorem [6,7], these two facts imply that compatibility can be decided in time linear in the size of the display graph. Unfortunately, Bryant and Lagergren’s argument amounts essentially to only an existential proof, as it is not clear how to obtain an explicit algorithm for unrooted compatibility from it.

A necessary step towards finding a practical algorithm for compatibility —and indeed for agreement— is to develop an explicit characterization of the problem. In earlier work [8], we made some progress in this direction, characterizing tree compatibility in terms of the existence of a legal triangulation of the display graph of the profile. Gysel et al. [9] provided an alternative characterization, based on a structure they call the edge label intersection graph (ELIG) (see Section ‘Display graphs and edge label intersection graphs’). Their formulation is in some ways simpler than that of [8], allowing Gysel et al. to express tree compatibility as a chordal sandwich problem. Neither [8] nor [9] deal with agreement.

Here, we show that the connection between separators in the ELIG and cuts in the display graph (explored in Section ‘Display graphs and edge label intersection graphs’) leads to a new, and natural, characterization of compatibility in terms of minimal cuts in the display graph (Section ‘Characterizing compatibility via cuts’). We then show how such cuts are closely related to the splits of the compatible supertree (Section ‘Splits and cuts’). Next, we give a characterization of the agreement in terms of minimal cuts of the display graph (Section ‘Characterizing agreement via cuts’). To our knowledge, there was no previous characterization of the agreement supertree problem for unrooted trees. Lastly, we examine the connection between the triangulation-based and the cut-based perspectives on compatibility (Section ‘Relationship to legal triangulations’).

## Preliminaries

### Splits, compatibility, and agreement

A *split* of a label set *L* is a bipartition of *L* consisting of non-empty sets. We denote a split {*X*,*Y*} by *X*|*Y*. A split is *non-trivial* if neither of its sets is a singleton; otherwise, it is *trivial*. Let *T* be a phylogenetic tree. Let *e* be an edge of *T*. Deletion of *e* disconnects *T* into two subtrees *T*_1_ and *T*_2_. If *L*_1_ and *L*_2_ denote the set of all labels in *T*_1_ and *T*_2_, respectively, then *L*_1_|*L*_2_ is a split of ℒ(T). We denote by *σ*_*e*_(*T*) the split corresponding to edge *e* of *T*; if *e* is a leaf edge, then *σ*_*e*_(*T*) is a trivial split. Let Σ(*T*) denote the set of all splits corresponding to internal edges of *T* and Σ_*t* *r* *i* *v*_(*T*) denote the set of all (trivial) splits corresponding to leaf edges of *T*.

A tree *Tdisplays* a split *X*|*Y* if there exists an internal edge *e* of *T* where *σ*_*e*_(*T*)=*X*|*Y*. A set of splits of a label set *L* is *compatible* if there exists a tree that displays all the splits in the set. It is well-known that two splits *A*_1_|*A*_2_ and *B*_1_|*B*_2_ are compatible if and only if at least one of *A*_1_∩*B*_1_, *A*_1_∩*B*_2_, *A*_2_∩*B*_1_ and *A*_2_∩*B*_2_ is empty [10]. Note that a trivial split of *L* is compatible with every split of *L*.

#### **Theorem 1** (Splits-Equivalence Theorem [10,11]).

Let Σ be a collection of splits of a label set *X* that includes all trivial splits. Then, Σ=Σ(*T*)∪Σ_*t* *r* *i* *v*_(*T*) for some phylogenetic tree *T* with label set *X* if and only if the splits in Σ are pairwise compatible. Tree *T* is unique up to isomorphism.

Let *S* be a phylogenetic tree and let *Y* be a subset of ℒ(S). Then, *S*_|*Y*_ denotes the tree obtained by suppressing any degree-two vertices in the minimal subtree of *S* connecting the labels in *Y*. Now, let *T* be a phylogenetic tree such that ℒ(T)⊆ℒ(S). Then, *SdisplaysT* if and only if $\Sigma (T)\subseteq \Sigma (S_{\left|ℒ(T\right)})$; *T* and *Sagree* if and only if $\Sigma (T)=\Sigma (S_{\left|ℒ(T\right)})$.

### Cliques, separators, cuts, and triangulations

Let *G* be a graph. We represent the vertices and edges of *G* by *V*(*G*) and *E*(*G*) respectively. A *clique* of *G* is a complete subgraph of *G*. A clique *H* of *G* is *maximal* if there is no other clique *H*^′^ of *G* where *V*(*H*)⊂*V*(*H*^′^). For any *U*⊆*V*(*G*), *G*−*U* is the graph derived by removing vertices of *U* and their incident edges from *G*. For any *F*⊆*E*(*G*), *G*−*F* is the graph with vertex set *V*(*G*) and edge set *E*(*G*) ∖ *F*.

For any two nonadjacent vertices *a* and *b* of *G*, an *a*-*bseparator* of *G* is a set *U* of vertices where *U*⊂*V*(*G*) and *a* and *b* are in different connected components of *G*−*U*. An *a*-*b* separator *U* is *minimal* if for every *U*^′^⊂*U*, *U*^′^ is not an *a*-*b* separator. A set *U*⊆*V*(*G*) is a *minimal separator* if *U* is a minimal *a*-*b* separator for some nonadjacent vertices *a* and *b* of *G*. We represent the set of all minimal separators of graph *G* by △_*G*_. Two minimal separators *U* and *U*^′^ are *parallel* if *G*−*U* contains at most one component *H* where *V*(*H*)∩*U*^′^≠∅.

A connected component *H* of *G*−*U* is *full* if for every *u*∈*U* there exists some vertex *v*∈*H* where {*u*,*v*}∈*E*(*G*).

#### **Lemma 1** ([12]).

For a graph *G* and any *U*⊂*V*(*G*), *U* is a minimal separator of *G* if and only if *G*−*U* has at least two full components.

A *chord* is an edge between two nonadjacent vertices of a cycle. A graph *H* is *chordal* if and only if every cycle of length four or greater in *H* has a chord. A chordal graph *H* is a *triangulation* of graph *G* if *V*(*G*)=*V*(*H*) and *E*(*G*)⊆*E*(*H*). The edges in *E*(*H*) ∖ *E*(*G*) are called *fill-in* edges of *G*. A triangulation is *minimal* if removing any fill-in edge yields a non-chordal graph.

A *clique tree* of a chordal graph *H* is a pair (*T*,*B*) where (i) *T* is a tree, (ii) *B* is a bijective function from vertices of *T* to maximal cliques of *H*, and (iii) for every vertex *v*∈*H*, the set of all vertices *x* of *T* where *v*∈*B*(*x*) induces a subtree in *T*. Property (iii) is called *coherence*.

Let  be a collection of subsets of *V*(*G*). We represent by $G_{F}$ the graph derived from *G* by making the set of vertices of *X* a clique for every X∈F. The next result summarizes basic facts about separators and triangulations (see [12-14]).

#### **Theorem 2**.

Let  be a maximal set of pairwise parallel minimal separators of *G* and *H* be a minimal triangulation of *G*. Then, the following statements hold. 

1. $G_{F}$ is a minimal triangulation of *G*.

2. Let (*T*,*B*) be a clique tree of $G_{F}$. There exists a minimal separator F∈F if and only if there exist two adjacent vertices *x* and *y* in *T* where *B*(*x*)∩*B*(*y*)=*F*.

3. △_*H*_ is a maximal set of pairwise parallel minimal separators of *G* and $G_{{△}_{H}}=H$.

A *cut* in a connected graph *G* is a subset *F* of edges of *G* such that *G*−*F* is disconnected. A cut *F* is *minimal* if there does not exist *F*^′^⊂*F* where *G*−*F*^′^ is disconnected. Note that if *F* is minimal, *G*−*F* has exactly be two connected components. Two minimal cuts *F* and *F*^′^ are *parallel* if *G*−*F* has at most one connected component *H* where *E*(*H*)∩*F*^′^≠∅.

## Display graphs and edge label intersection graphs

We now introduce the two main notions that we use to characterize compatibility and agreement: the display graph and the edge label intersection graph. We then present some known results about these graphs, along with new results on the relationships between them. Here and in the rest of the paper, [*m*] denotes the set {1,…,*m*}, where *m* is a positive integer. Since for any phylogenetic tree *T* there is a bijection between the leaves of *T* and ℒ(T), we refer to the leaves of *T* by their labels.

**Figure 1:**
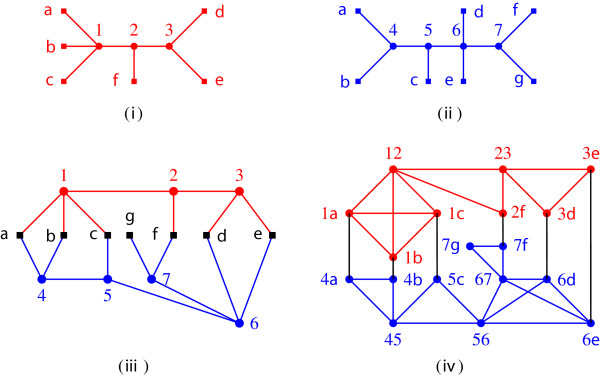
**Compatible trees. (i)** First input tree. **(ii)** A second input tree, compatible with the first. **(iii)** Display graph of the input trees. **(iv)** Edge label intersection graph of the input trees; for each vertex, *uv* represents edge {*u*,*v*} of the display graph.

Let $P=\{T_{1},T_{2},\cdots \;,T_{k}\}$ be a profile. We assume that for any *i*,*j*∈[*k*] such that *i*≠*j*, the sets of internal vertices of input trees *T*_*i*_ and *T*_*j*_ are disjoint. The *display graph* of , denoted by G(P), is a graph whose vertex set is $\underset{i\in [k]}{⋃}V(T_{i})$ and edge set is $\underset{j\in [k]}{⋃}E(T_{j})$ (see Figure 1). A vertex *v* of G(P) is a *leaf* if v∈ℒ(P). Every other vertex of G(P) is an *internal*. An edge of G(P) is *internal* if its endpoints are both internal.

A triangulation *G*^′^ of G(P) is *legal* if it satisfies the following conditions. 

1. For every clique *C* of *G*^′^, if *C* contains an internal edge, then it contains no other edge of G(P).

2. No fill-in edge in *G*^′^ has a leaf as an endpoint.

### **Theorem 3** (Vakati, Fernández-Baca [8]).

A profile  of unrooted phylogenetic trees is compatible if and only if G(P) has a legal triangulation.

In what follows, we assume that G(P) is connected. If it is not, the connected components of G(P) induce a partition of  into sub-profiles such that for each sub-profile $P^{\prime }$, $G(P^{\prime })$ is a connected component of G(P). It is easy to see that  is compatible if and only if each sub-profile is compatible.

The *edge label intersection graph of*, denoted LG(P), is the line graph of G(P)[9]. That is, the vertex set of LG(*G*) is E(G(P)) and two vertices of LG(P) are adjacent if the corresponding edges in G(P) share an endpoint. (We should note that Gysel et al. [9] refer to LG(P) as the modified edge label intersection graph.) For an unrooted tree *T*, LG(*T*) denotes LG({*T*}).

### **Observation 1**.

Let *F* be a set of edges of G(P) and let $\left\{v_{1},v_{2},\ldots ,v_{m}\}\subseteq V(G(P)\right)$ where *m*≥2. Then, *v*_1_,*v*_2_,…,*v*_*m*_ is a path in G(P)−F if and only if {*v*_1_,*v*_2_},…,{*v*_*m*−1_,*v*_*m*_} is a path in in LG(P)−F.

Thus, if G(P) is connected, so is LG(P). Hence, in what follows, we assume that LG(P) is connected.

A fill-in edge for LG(P) is *valid* if for every T∈P, at least one of the endpoints of the edge is not in LG(*T*). A triangulation *H* of LG(P) is *restricted* if every fill-in edge of *H* is valid.

### **Theorem 4** (Gysel et al. [9]).

A profile  of unrooted phylogenetic trees is compatible if and only if LG(P) has a restricted triangulation.

A minimal separator *F* of LG(P) is *legal* if for every T∈P, all the edges of *T* in *F* share a common endpoint; i.e., *F*∩*E*(*T*) is a clique in LG(*T*). The following theorem was mentioned in [9]. For future reference, we formally state it and prove it here.

### **Theorem 5**.

A profile  is compatible if and only if there exists a maximal set  of pairwise parallel minimal separators in LG(P) where every separator in  is legal.

### *Proof*.

Our approach is similar to the one used by Gusfield in [15]. Assume that  is compatible. From Theorem 4, there exists a restricted triangulation *H* of LG(P). We can assume that *H* is minimal (if it is not, simply delete fill-in edges repeatedly from *H* until it is minimal). Let $F={△}_{H}$. From Theorem 2,  is a maximal set of pairwise parallel minimal separators of LG(P) and $\text{LG}{\left(P\right)}_{F}=H$. Suppose  contains a separator *F* that is not legal. Let {*e*,*e*^′^}⊆*F* where {*e*,*e*^′^}⊆*E*(*T*) for some input tree *T* and *e*∩*e*^′^=∅. The vertices of *F* form a clique in *H*. Thus, *H* contains the edge {*e*,*e*^′^}. Since {*e*,*e*^′^} is not a valid edge, *H* is not a restricted triangulation, a contradiction. Hence, every separator in  is legal.

Let  be a maximal set of pairwise parallel minimal separators of LG(P) where every separator in  is legal. From Theorem 2, $\text{LG}{\left(P\right)}_{F}$ is a minimal triangulation of LG(F). If $\left\{e,e^{\prime }\}\in E(\text{LG}{\left(P\right)}_{F}\right)$ is a fill-in edge, then *e*∩*e*^′^=∅ and there exists a minimal separator F∈F where {*e*,*e*^′^}⊆*F*. Since *F* is legal, if {*e*,*e*^′^}⊆*E*(*T*) for some input tree *T* then *e*∩*e*^′^≠∅. Thus, *e* and *e*^′^ are not both from LG(*T*) for any input tree *T*. Hence, every fill-in edge in $\text{LG}{\left(P\right)}_{F}$ is valid, and $\text{LG}{\left(P\right)}_{F}$ is a restricted triangulation.

Let *u* of be a vertex of some input tree, We write Inc(*u*) to denote the set of all edges of G(P) incident on *u*. Equivalently, Inc(*u*) is the set of all vertices *e* of LG(P) such that *u*∈*e*.

Let *F* be a cut of the display graph G(P). *F* is *legal* if for every tree T∈P, the edges of *T* in *F* are incident on a common vertex; i.e., if *F*∩*E*(*T*)⊆Inc(*u*) for some *u*∈*V*(*T*). *F* is *nice* if *F* is legal and each connected component of G(P)−F has at least one edge.

### **Lemma 2**.

Let *F* be a subset of E(G(P)). Then, *F* is a legal minimal separator of LG(P) if and only if *F* is a nice minimal cut of G(P).

To prove the Lemma 2, we need two auxiliary lemmas and a corollary.

### **Lemma 3**.

Let *F* be any minimal separator of LG(P) and *u* be any vertex of any input tree. Then, Inc(*u*)⫅̸*F*.

### *Proof*.

Suppose *F* is a minimal *a*-*b* separator of LG(P) and *u* is a vertex of some input tree such that Inc(*u*)⊆*F*. Consider any vertex *e*∈Inc(*u*). Then, there exists a path *π* from *a* to *b* in LG(P) where *e* is the only vertex of *F* in *π*. If such a path *π* did not exist, then *F*−*e* would still be an *a*-*b* separator, and *F* would not be minimal, a contradiction. Let *e*_1_ and *e*_2_ be the neighbors of *e* in *π* and let *e*={*u*,*v*}. Since Inc(*u*)⊆*F*, *π* does not contain any other vertex *e*^′^ where *u*∈*e*^′^. Thus, *e*∩*e*_1_={*v*} and *e*∩*e*_2_={*v*}. Let *π*=*a*,…,*e*_1_,*e*,*e*_2_,…,*b*. Then *π*^′^=*a*,…,*e*_1_,*e*_2_,…,*b* is also a path from *a* to *b*. But *π*^′^ does not contain any vertex of *F*, contradicting the assumption that *F* is a separator of LG(P). Hence, neither such a minimal separator *F* nor such a vertex *u* exist.

### **Lemma 4**.

If *F* is a minimal separator of LG(P), then LG(P)−F has exactly two connected components.

### *Proof*.

Assume that LG(P)−F has more than two connected components. By Lemma 1, LG(P)−F has at least two full components. Let *H*_1_ and *H*_2_ be two full components of LG(P)−F. Let *H*_3_ be a connected component of LG(P)−F different from *H*_1_ and *H*_2_. By assumption LG(P) is connected. Thus, there exists an edge {*e*,*e*_3_} in LG(P) where *e*∈*F* and *e*_3_∈*H*_3_. Since *H*_1_ and *H*_2_ are full components, there exist edges {*e*,*e*_1_} and {*e*,*e*_2_} in LG(P) where *e*_1_∈*V*(*H*_1_) and *e*_2_∈*V*(*H*_2_).

Let *e*={*u*,*v*}, and assume without loss of generality that *u*∈*e*∩*e*_3_. Then, there is no vertex *f*∈*V*(*H*_1_) where *u*∈*e*∩*f*. Thus, *v*∈*e*∩*e*_1_. Similarly, there is no vertex *f*∈*V*(*H*_2_) such that *u*∈*f*∩*e* or *v*∈*f*∩*e*. But then *H*_2_ does not contain a vertex adjacent to *e*, so *H*_2_ is not a full component, a contradiction.

### **Corollary 1**.

If *F* is a minimal separator of LG(P), then $\text{LG}(P)-F^{\prime }$ is connected for any *F*^′^⊂*F*.

### *Proof of Lemma 2*.

We prove that if *F* is a legal minimal separator of LG(P) then *F* is a nice minimal cut of G(P). The proof for the other direction is similar and is omitted.

First, we show that *F* is a cut of G(P). Assume the contrary. Let {*u*,*v*} and {*p*,*q*} be vertices in different components of LG(P)−F. Since G(P)−F is connected, there is a path between vertices *u* and *q* in this graph. Also, {*u*,*v*}∉*F* and {*p*,*q*}∉*F*. Thus, by Observation 1 there is also a path between vertices {*u*,*v*} and {*p*,*q*} of LG(P)−F. This implies that {*u*,*v*} and {*p*,*q*} are in the same connected component of LG(P)−F, a contradiction. Thus *F* is a cut.

Next we show that *F* is a nice cut of G(P). For every T∈P all the vertices of LG(*T*) in *F* form a clique in LG(*T*). Thus, all the edges of *T* in *F* are incident on a common vertex, so *F* is legal. To complete the proof, assume that G(P)−F has a connected component with no edge and let *u* be the vertex in one such component. Then, Inc(*u*)⊆*F*. But *F* is a minimal separator of LG(P), and by Lemma 3, Inc(*u*) ⊈ *F*, a contradiction. Thus, *F* is a nice cut.

Lastly, we show that *F* is a minimal cut of G(P). Assume, on the contrary, that there exists *F*^′^⊂*F* where $G(P)-F^{\prime }$ is disconnected. Since *F*^′^⊂*F* and every connected component of G(P)−F has at least one edge, every connected component of $G(P)-F^{\prime }$ also has at least one edge. Let {*u*,*v*} and {*p*,*q*} be the edges in different components of $G(P)-F^{\prime }$. By Corollary 1, $\text{LG}(P)-F^{\prime }$ is connected and thus, there is a path between {*u*,*v*} and {*p*,*q*} in $\text{LG}(P)-F^{\prime }$. By Observation 1 there must also be a path between vertices *u* and *p* in $G(P)-F^{\prime }$. Hence, edges {*u*,*v*} and {*p*,*q*} are in the same connected component of *G*−*F*^′^, a contradiction. Thus, *F* is a minimal cut.

### **Lemma 5**.

Two legal minimal separators *F* and *F*^′^ of LG(P) are parallel if and only if the nice minimal cuts *F* and *F*^′^ are parallel in G(P).

### *Proof*.

Assume that separators *F* and *F*^′^ of LG(P) are parallel, but cuts *F* and *F*^′^ of G(P) are not. Then, there exists a set {{*u*,*v*},{*p*,*q*}}⊆*F*^′^ where {*u*,*v*} and {*p*,*q*} are in different components of G(P)−F. Since *F* and *F*^′^ are parallel separators in LG(P), and *F* does not contain {*u*,*v*} and {*p*,*q*}, there exists a path between vertices {*u*,*v*} and {*p*,*q*} in LG(P)−F. Then, by Observation 1 there also exists a path between vertices *u* and *q* in G(P)−F. Thus, {*u*,*v*} and {*p*,*q*} are in the same connected component of G(P)−F, a contradiction.

The other direction can be proved similarly, using Observation 1.

The next lemma, from [9], follows from the definition of restricted triangulation.

### **Lemma 6**.

Let *H* be a restricted triangulation of LG(P) and let (*T*,*B*) be a clique tree of *H*. Let *e*={*u*,*v*} be any vertex in LG(P). Then, there does not exist a node *x*∈*V*(*T*) where *B*(*x*) contains vertices from both Inc(*u*) ∖ *e* and Inc(*v*) ∖ *e*.

### **Lemma 7**.

Let *T* be a tree in  and suppose *F* is a minimal cut of G(P) that contains precisely one edge *e* of *T*. Then, the edges of the two subtrees of *T*−*e* are in different connected components of G(P)−F.

### *Proof*.

Let *e*={*u*,*v*}. For each *x*∈*e*, let *T*_*x*_ denote the subtree containing vertex *x* in *T*−*e*. For each vertex *x*∈*e*, all the edges of *T*_*x*_ are in the same connected component of G(P)−F as *x*, because *e* is the only edge of *T* in *F*. Since *F* is a minimal cut of G(P), the endpoints of *e* are in different connected components of G(P)−F. Hence, the edges of *T*_*u*_ and *T*_*v*_ are also in different connected components of G(P)−F.

## Characterizing compatibility via cuts

A set  of cuts of G(P) is *complete* if, for every input tree T∈P and every internal edge *e* of *T*, there is a cut F∈F where *e* is the only edge of *T* in *F*.

### **Lemma 8**.

G(P) has a complete set of pairwise parallel nice minimal cuts if and only if it has a complete set of pairwise parallel legal minimal cuts.

### *Proof*.

The “only if part” follows from the definition of a nice cut. Let  be a complete set of pairwise parallel legal minimal cuts. Consider any minimal subset $F^{\prime }$ of  that is also complete. Let *F* be a legal minimal cut of $F^{\prime }$. Since $F^{\prime }$ is minimal, there exists an edge *e*∈*F* of some input tree *T* such that *e* is the only edge of *T* in *F*. Also, since *e* is an internal edge, both subtrees of *T*−*e* have at least one edge each. Thus by Lemma 7, both connected components of G(P)−F have at least one edge each. Hence, *F* is a nice minimal cut of G(P). It follows that $F^{\prime }$ is a complete set of pairwise parallel nice minimal cuts of G(P).

We now characterize the compatibility of a profile in terms of minimal cuts in the display graph of the profile.

### **Theorem 6**.

A profile  of unrooted phylogenetic trees is compatible if and only if there exists a complete set of pairwise parallel legal minimal cuts for G(P).

### **Example 1**.

For the display graph of Figure 1, let $F=\{F_{1},F_{2},F_{3},F_{4}\}$, where *F*_1_={{1,2},{5,6}}, *F*_2_={{2,3},{6,7},{5,6}}, *F*_3_={{4,5},{1,2},{1,*c*}} and *F*_4_={{6,7},{2,*f*}}. Then,  is a complete set of pairwise parallel nice minimal cuts.

Theorem 6 has an analog in terms of LG(P). Let us say that a set  of legal minimal separators of LG(P) is *complete* if for every internal edge *e* of an input tree *T*, there exists a separator F∈F where *e* is the only vertex of LG(*T*) in *F*.

### **Theorem 7**.

A profile  of unrooted phylogenetic trees is compatible if and only if there exists a complete set of pairwise parallel legal minimal separators for LG(P).

This result is a direct consequence of Theorem 6 and Lemmas 2, 5, and 8, so we omit its proof. Instead, we focus on the proof of Theorem 6, for which we need the next fact.

### **Lemma 9**.

The following two statements are equivalent. 

1. There exists a maximal set  of pairwise parallel minimal separators of LG(P) where every separator in  is legal.

2. There exists a complete set of pairwise parallel nice minimal cuts for G(P).

### *Proof*.

*(i) ⇒ (ii):* We show that for every internal edge *e*={*u*,*v*} of an input tree *T* there exists a minimal separator in  that contains only vertex *e* from LG(*T*). Then it follows from Lemmas 2 and 5 that  is a complete set of pairwise parallel nice minimal cuts for G(P).

As shown in the proof of Theorem 5, $\text{LG}{\left(P\right)}_{F}$ is a restricted minimal triangulation of LG(P). Let (*S*,*B*) be a clique tree of $\text{LG}{\left(P\right)}_{F}$. By definition, the vertices in each of the sets Inc(*u*) and Inc(*v*) form a clique in LG(P). Consider any vertex *p* of *S* where Inc(*u*)⊆*B*(*p*) and any vertex *q* of *S* where Inc(*v*)⊆*B*(*q*). (Since (*S*,*B*) is a clique tree of $\text{LG}{\left(P\right)}_{F}$, such vertices *p* and *q* must exist.) Also, by Lemma 6, *p*≠*q*, *B*(*p*)∩(Inc(*v*) ∖ {*e*})=∅ and *B*(*q*)∩(Inc(*u*) ∖ {*e*})=∅.

Let *π*=*p*,*x*_1_,*x*_2_,…,*x*_*m*_,*q* be the path from *p* to *q* in *S* where *m*≥0. Let *x*_0_=*p* and *x*_*m*+1_=*q*. Let *x*_*i*_ be the vertex nearest to *p* in path *π* where *i*∈[*m*+1] and *B*(*x*_*i*_)∩(Inc(*u*) ∖ {*e*})=∅. Let *F*=*B*(*x*_*i*−1_)∩*B*(*x*_*i*_). Then by Theorem 2, F∈F. Since Inc(*u*)∩Inc(*v*)={*e*}, by the coherence property, *e*∈*B*(*x*_*j*_) for every *j*∈[*m*]. Thus, *e*∈*F*. By Lemma 6, *B*(*x*_*i*−1_)∩(Inc(*v*) ∖ {*e*})=∅. Since *B*(*x*_*i*_)∩(Inc(*u*) ∖ {*e*})=∅, *F*∩Inc(*u*)={*e*} and *F*∩Inc(*v*)={*e*}. Thus, for every vertex *e*^′^∈LG(*T*) where *e*≠*e*^′^ and *e*∩*e*^′^≠∅, *e*^′^∉*F*. Also, since every separator in  is legal, we have *f*∉*F* for every vertex *f*∈LG(*T*) where *f*∩*e*=∅. Thus, *e* is the only vertex of LG(*T*) in *F*.

*(i) ⇐ (ii):* Consider any complete set of pairwise parallel nice minimal cuts $F^{\prime }$ of G(P). By Lemmas 2 and 5, $F^{\prime }$ is a set of pairwise parallel legal minimal separators of LG(P). There exists a maximal set  of pairwise parallel minimal separators where $F^{\prime }\subseteq F$.

Assume that $F\;\setminus \;F^{\prime }$ contains a minimal separator *F* that is not legal. Then, there must exist a tree T∈P where at least two nonincident edges *e*_1_={*x*,*y*} and *e*_2_={*x*^′^,*y*^′^} of *T* are in *F*. Consider any internal edge *e*_3_ in *T* where *e*_1_ and *e*_2_ are in different components of *T*−*e*_3_. Such an edge exists because *e*_1_ and *e*_2_ are nonincident. Since $F^{\prime }$ is complete, there exists a cut $F^{\prime }\in F^{\prime }$ where *e*_3_ is the only edge of *T* in *F*^′^. Since *F* and *F*^′^ are in , they are parallel to each other and vertices *e*_1_ and *e*_2_ are in the same connected component of $\text{LG}(P)-F^{\prime }$. Thus, by Observation 1, there exists a path between vertices *x* and *x*^′^ in $G(P)-F^{\prime }$ and edges *e*_1_ and *e*_2_ are also in the same connected component of $G(P)-F^{\prime }$. But by Lemma 7 that is impossible.

Thus, every separator of $F\;\setminus \;F^{\prime }$ is legal and  is a maximal set of pairwise minimal separators of LG(P) where every separator in  is legal.

### *Proof of Theorem 6*.

By Theorem 5 and Lemma 9, profile  is compatible if and only if there exists a complete set of pairwise parallel nice minimal cuts for G(P). The rest follows from Lemma 8.

## Splits and cuts

We first argue that for every nice minimal cut of G(P) we can derive a split of ℒ(P). We use the following notation: if *H* is a subgraph of G(P), then ℒ(H) represents the set of all leaves of *H*

### **Lemma 10**.

Let *F* be a nice minimal cut of G(P) and let *G*_1_ and *G*_2_ be the two connected components of G(P)−F. Then, *L*(*G*_*i*_)≠∅ for *i*∈{1,2}. In particular, $ℒ(G_{1})|ℒ(G_{2})$ is a split of ℒ(P).

### *Proof*.

Consider *G*_*i*_ for each *i*∈{1,2}. We show that $ℒ(G_{i})$ is non-empty. Since *F* is nice, *G*_*i*_ contains at least one edge *e* of G(P). If *e* is a non-internal edge, then $ℒ(G_{i})$ is non-empty. Assume that *e*={*u*,*v*} is an internal edge of some input tree *T*. If *F* does not contain an edge of *T*, then $ℒ(T)\subseteq ℒ(G_{i})$ and thus $ℒ(G_{i})$ is non-empty. Assume that *F* contains one or more edges of *T*. Let *T*_*u*_, *T*_*v*_ be the two subtrees of *T*−*e*. Since *F* is a nice minimal cut, *F* contains edges from either *T*_*u*_ or *T*_*v*_ but not both. Without loss of generality assume that *F* does not contain edges from *T*_*u*_. Then, every edge of *T*_*u*_ is in the same component as *e*. Since *T*_*u*_ contains at least one leaf, $ℒ(G_{i})$ is non-empty. Thus, $ℒ(G_{1})|ℒ(G_{2})$ is a split of ℒ(P).

Let *σ*(*F*) denote the split of ℒ(P) induced by a nice minimal cut *F*. If  is a set of nice minimal cuts of G(P), Σ(F) denotes the set of all the non-trivial splits in $\underset{F\in F}{⋃}\sigma (F)$. The following result expresses the relationship between complete sets of nice minimal cuts and the compatibility of splits.

### **Theorem 8**.

If G(P) has a complete set of pairwise parallel nice minimal cuts , then Σ(F) is compatible and any compatible tree for Σ(F) is also a compatible tree for .

### **Example 2**.

For the complete set of pairwise parallel nice minimal cuts $F=\{F_{1},F_{2},F_{3},F_{4}\}$ for the display graph of Example 1, we have *σ*(*F*_1_)=*abc*|*defg*, *σ*(*F*_2_)=*abcfg*|*de*, *σ*(*F*_3_)=*ab*|*cdefg*, and *σ*(*F*_4_)=*abcde*|*fg*. Note that these splits are pairwise compatible.

The proof of Theorem 8 uses the following lemma.

### **Lemma 11**.

Let *F*_1_ and *F*_2_ be two parallel nice minimal cuts of G(P). Then, *σ*(*F*_1_) and *σ*(*F*_2_) are compatible.

### *Proof*.

Let *σ*(*F*_1_)=*U*_1_|*U*_2_ and *σ*(*F*_2_)=*V*_1_|*V*_2_. Assume that *σ*(*F*_1_) and *σ*(*F*_2_) are incompatible. Thus, *U*_*i*_∩*V*_*j*_≠∅ for every *i*,*j*∈{1,2}. Let *a*∈*U*_1_∩*V*_1_, *b*∈*U*_1_∩*V*_2_, *c*∈*U*_2_∩*V*_1_ and *d*∈*U*_2_∩*V*_2_. Since {*a*,*b*}⊆*U*_1_, there exists a path *π*_1_ between leaves *a* and *b* in $G(P)-F_{1}$. But *a* and *b* are in different components of $G(P)-F_{2}$. Thus, an edge *e*_1_ of path *π*_1_ is in the cut *F*_2_. Similarly, {*c*,*d*}⊆*U*_2_ and there exists a path *π*_2_ between labels *c* and *d* in $G(P)-F_{1}$. Since *c* and *d* are in different components of $G(P)-F_{2}$, cut *F*_2_ contains an edge *e*_2_ of path *π*_2_. But *π*_1_ and *π*_2_ are in different components of $G(P)-F_{1}$, so edges *e*_1_ and *e*_2_ are in different components of $G(P)-F_{1}$. Since {*e*_1_,*e*_2_}⊆*F*_2_, the cuts *F*_1_ and *F*_2_ are not parallel, a contradiction.

### *Proof of Theorem 8*.

The compatibility of Σ(F) follows from Lemma 11 and Theorem 1. Let *S* be a compatible tree for Σ(*F*), let *T* be an input tree of , let $S^{\prime }=S_{\left|ℒ(T\right)}$, and let *e* be any internal edge of *T*. We show that *S*^′^ displays *σ*(*e*)

Let *σ*(*e*)=*A*|*B*. There exists a cut F∈F where *e* is the only edge of *T* in *F*. By Lemma 7, since *F* is minimal, the leaves of sets *A* and *B* are in different components of G(P)−F. Thus, if *σ*(*F*)=*A*^′^|*B*^′^ then, up to renaming of sets, we have *A*⊆*A*^′^ and *B*⊆*B*^′^. Because *S* displays *σ*(*F*), *S*^′^ also displays *σ*(*e*). Since *S*^′^ displays all the splits of *T*, *T* can be obtained from *S*^′^ by contracting zero or more edges [10]. Thus, *S* displays *T*. Since *S* displays every tree in , *S* is a compatible tree for .

## Characterizing agreement via cuts

The following characterization of agreement is similar to the one for tree compatibility given by Theorem 6, except for an additional restriction on the minimal cuts.

### **Theorem 9**.

A profile  has an agreement supertree if and only if G(P) has a complete set  of pairwise parallel legal minimal cuts where, for every cut F∈F and for every T∈P, there is at most one edge of *T* in *F*.

### **Example 3**.

**Figure 2:**
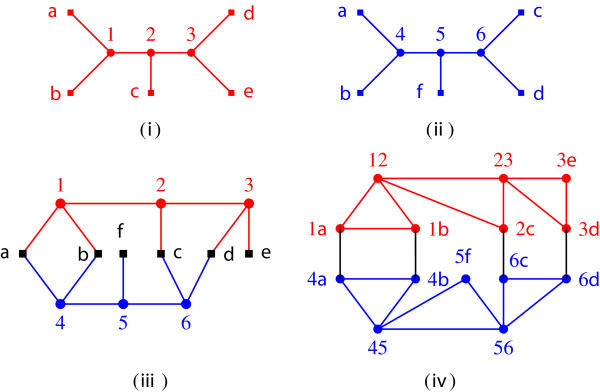
**Agreeing trees.(i)** First input tree. **(ii)** Second input tree, which agrees with the first. **(iii)** Display graph of the input trees. **(iv)** Edge label intersection graph of the input trees, where label *uv* represents edge {*u*,*v*} of the display graph.

One can verify that the display graph of Figure 1 does not meet the conditions of Theorem 9 and, thus, the associated profile does not have an AST. On the other hand, for the display graph of Figure 2, let $F=\{F_{1},F_{2},F_{3}\}$, where *F*_1_={{1,2},{4,5}}, *F*_2_={{1,2},{5,6}} and *F*_3_={{2,3},{6,*d*}}. For any given input tree *T*, every cut in  has at most one edge of *T*. Also,  is a complete set of pairwise parallel legal minimal cuts. Thus, by Theorem 9, the input trees of Figure 2 have an AST

The analogue of Theorem 9 for LG(P) stated next follows from Theorem 9 and Lemmas 2, 5, and 8.

### **Theorem 10**.

A profile  has an agreement supertree if and only if LG(P) has a complete set  of pairwise parallel legal minimal separators where, for every F∈F and every T∈P, there is at most one vertex of LG(*T*) in *F*.

Theorem 9 follows from Lemma 8 and the next result.

### **Lemma 12**.

A profile  has an agreement supertree if and only if G(P) has a complete set  of pairwise parallel nice minimal cuts where, for every cut F∈F and every T∈P, there is at most one edge of *T* in *F*.

The rest of the section is devoted to the proof of Lemma 12

Let *S* be an AST of  and let *e*={*u*,*v*} be an edge of *S*. Let *S*_*u*_ and *S*_*v*_ be the subtrees of *S*−*e* containing *u* and *v*, respectively. Let $L_{u}=ℒ(S_{u})$ and $L_{v}=ℒ(S_{v})$. Thus, *σ*_*e*_(*S*)=*L*_*u*_|*L*_*v*_. Assume that there exists an input tree *T* where $ℒ(T)\cap L_{x}\neq \emptyset$ for each *x*∈{*u*,*v*}. Then there exists an edge *f*∈*E*(*T*) where, if *σ*_*f*_(*T*)=*A*_1_|*A*_2_, then *A*_1_⊆*L*_*u*_ and *A*_2_⊆*L*_*v*_. (If there were no such edge, $S_{\left|ℒ(T\right)}$ would contain a split that is not in *T* and would thus not be isomorphic to *T*.) We call *e* an *agreement edge* of *S* corresponding to edge *f* of *T*. Note that there does not exist any other edge *f*^′^ of *T* where *e* is also an agreement edge of *S* with respect to edge *f*^′^ of *T*.

The *cut function* of an AST *S* of  is the mapping *Ψ* from *E*(*S*) to subsets of edges of G(P) defined as follows. For every *e*∈*E*(*S*), an edge *f* of an input tree *T* is in *Ψ*(*e*) if and only if *e* is an agreement edge of *S* corresponding to edge *f* of *T*. Observe that *Ψ* is uniquely defined. Given an edge *e*∈*E*(*S*), we define a set *V*_*x*_ for each *x*∈*e* as follows. For every T∈P, let *V*_*x*,*T*_ consist of all the vertices of the minimal subtree of *T* connecting the labels in $ℒ(T)\cap L_{x}$. Then, $V_{x}=\underset{T\in P}{⋃}V_{x,T}$. Note that if *e*={*u*,*v*} then {*V*_*u*_,*V*_*v*_} is a partition of V(G(P)).

### **Lemma 13**.

Let *S* be an AST of  and let *Ψ* be the cut function of *S*. Then, for every edge *e*∈*E*(*S*), 

(i) *Ψ*(*e*) is a cut of G(P) and

(ii) *Ψ*(*e*) is a minimal cut of G(P) if and only if G(P)−Ψ(e) has exactly two connected components.

### *Proof*.

*(i)* Let *e*={*u*,*v*}. We show that G(P)−Ψ(e) does not contain an edge whose endpoints are in distinct sets of {*V*_*u*_,*V*_*v*_}. Assume the contrary. Let *f*={*x*,*y*} be an edge of G(P)−Ψ(e) where *x*∈*V*_*u*_ and *y*∈*V*_*v*_.

Since f∈G(P)−Ψ(e), *f*∉*Ψ*(*e*). Suppose *f* is an edge of input tree *T*. There are two cases. 

1. *Ψ*(*e) does not contain an edge of T.* Then, there exists an endpoint *p* of *e* where $ℒ(T)\subseteq L_{p}$. Without loss of generality, let *u*=*p*. Then, *V*(*T*)⊆*V*_*u*_ and thus *y*∈*V*_*u*_, a contradiction.

2. *Ψ*(*e) contains an edgef*^′^≠*fof T.* Let *f*^′^={*r*,*s*} and let *L*_*r*_⊆*L*_*u*_ and *L*_*s*_⊆*L*_*v*_. Let *x*,*r* be the vertices of *f* and *f*^′^ where *L*_*x*_⊂*L*_*r*_. Since *T* is a phylogenetic tree, such vertices *x* and *r* exist. Since *L*_*r*_⊆*L*_*u*_, both the endpoints of *f* are in *V*_*u*_, a contradiction.

Thus, G(P)−Ψ(e) does not contain an edge whose endpoints are in different sets of {*V*_*u*_,*V*_*v*_}. Since *V*_*u*_ and *V*_*v*_ are non-empty, *Ψ*(*e*) is a cut of G(P).

*(ii)* The “only if” part follows from the definition of a minimal cut. We now prove the “if” part. Let *e*={*u*,*v*}. Assume that G(P)−Ψ(e) has exactly two connected components. From the proof of (*i*), *V*_*u*_ and *V*_*v*_ are the vertex sets of those two connected components. Consider any edge *f*∈*Ψ*(*e*). The endpoints of *f* are in different sets of {*V*_*u*_,*V*_*v*_} and thus are in different connected components of G(P)−Ψ(e). Hence, G(P)−(Ψ(e)∖{f}) is connected. Thus, if G(P)−Ψ(e) has exactly two connected components, *Ψ*(*e*) is a minimal cut of G(P).

The next observation summarizes two basic facts about cut functions.

### **Observation 2**.

Let *S* be an AST of . Then, the cut function *Ψ* of *S* has the following properties. 

1. For any two distinct edges *e*_1_ and *e*_2_ in *E*(*S*), *Ψ*(*e*_1_)≠*Ψ*(*e*_2_).

2. Let *e*={*u*,*v*} be an edge of *S*. For any input tree *T* where $ℒ(T)\cap L_{v}\neq \emptyset$, all the labels of $ℒ(T)\cap L_{v}$ are in the same connected component of G(P)−Ψ(e).

Let *S* be an AST of  and let *e* be an edge of *S*. Although Lemma 13 shows that *Ψ*(*e*) is a cut of G(P), *Ψ*(*e*) may not be minimal. We now argue that we can always construct an agreement supertree whose cut function gives minimal cuts

### **Lemma 14**.

If  has an AST, then it has an AST *S* of  whose cut function *Ψ* satisfies the following: For every edge *e*∈*S*, *Ψ*(*e*) is a minimal cut of G(P).

We prove Lemma 14 by arguing that any AST that fails to satisfy the required cut minimality property can be transformed into one that does, through repeated application of the “splitting” operation, defined next.

Suppose *e*=(*u*,*v*) is a an edge of *S* where *Ψ*(*e*) is not minimal. Let {*L*_1_,…,*L*_*m*_} be the partition of *L*_*v*_ where for every *i*∈[*m*], $L_{i}=ℒ(C)\cap L_{v}$ for some connected component *C* in G(P)−Ψ(e). We assume without loss of generality that *m*>1 (if not, we can just exchange the roles of *u* and *v*). Let *R*_*v*_ be the rooted tree derived from *S*_*v*_ by distinguishing vertex *v* as the root. Let *R*_*v*,*i*_ be the (rooted) tree obtained from the minimal subtree of *R*_*v*_ connecting the labels in *L*_*i*_ by distinguishing the vertex closest to *v* as the root and suppressing every other vertex that has degree two. To *split edge e at u* is to construct a new tree *S*^′^ from *S* in two steps: (i) delete the vertices of *R*_*v*_ from *S* and (ii) for every *i*∈[*m*], add an edge from *u* to the root of *R*_*v*,*i*_.

### **Observation 3**.

Let *S* be an AST of  and let *Ψ* be the cut function of *S*. Let *S*^′^ be the tree derived by splitting edge *e*={*u*,*v*} at *u*. Consider any connected component *C* of G(P)−Ψ(e) where $ℒ(C)\cap L_{v}\neq \emptyset$. Then, for every $X\subseteq (ℒ(C)\cap L_{v})$, *S*_|*X*_ and $S_{|X}^{\prime }$ are isomorphic.

The next observation follows from the definition of AST.

### **Observation 4**.

Let *S* and *T* be two phylogenetic trees where ℒ(T)⊆ℒ(S) and *T* agrees with *S*. Then, *T* and *S*_|*U*_ agree for every *U* such that ℒ(T)⊆U⊆ℒ(S).

### **Lemma 15**.

Let *S* be an AST of  and let *e*={*u*,*v*} be an edge of *S*. Let *S*^′^ be the tree derived by splitting edge *e* at *u*. Then, *S*^′^ is an AST of .

### *Proof*.

By construction, *S*^′^ is a phylogenetic tree over ℒ(P). As before, let {*L*_1_,…,*L*_*m*_} be the partition of *L*_*v*_ where for every *i*∈[*m*], $L_{i}=ℒ(C)\cap L_{v}$ for some connected component *C* in G(P)−Ψ(e). Consider any input tree *T* of profile . We prove that *T* and *S*^′^ agree. There are three cases. *Case 1:*$ℒ(T)\subseteq L_{u}$*.* Since $ℒ(T)\subseteq L_{u}$, by Observation 4, *T* and $S_{|L_{u}}$ agree. By the definition of the split operation, trees $S_{|L_{u}}$ and $S_{|L_{u}}^{\prime }$ are isomorphic. Thus, *T* and *S*^′^ agree. *Case 2:*$ℒ(T)\subseteq L_{v}$*.* By Observation 2(ii), $ℒ(T)\subseteq L_{i}$ for some *i*∈[*m*]. Since *T* and *S* agree and $ℒ(T)\subseteq L_{i}$, by Observation 4, *T* and $S_{|L_{i}}$ agree. By construction, trees $S_{|L_{i}}$ and $S_{|L_{i}}^{\prime }$ are isomorphic. Thus, *T* and *S*^′^ agree. *Case 3:*$\left(ℒ(T)\cap L_{u}\neq \emptyset \right)$ and $\left(ℒ(T)\cap L_{v}\neq \emptyset \right)$*.* By Observation 2(ii), $ℒ(T)\cap L_{v}\subseteq L_{i}$ for some *i*∈[*m*]. Since *T* and *S* agree and $ℒ(T)\subseteq (L_{u}\cup L_{i})$, by Observation 4, *T* also agrees with $S_{\left|(L_{u}\cup L_{i}\right)}$. By construction, trees $S_{\left|(L_{u}\cup L_{i}\right)}$ and $S_{\left|(L_{u}\cup L_{i}\right)}^{\prime }$ are isomorphic. Thus, *T* and $S_{\left|(L_{u}\cup L_{i}\right)}^{\prime }$ agree. It follows that *T* and *S*^′^ agree

Thus, *S*^′^ is an AST of .

Observe that if *S*^′^ is the tree obtained by splitting edge *e*={*u*,*v*} of *S* at *u*, then the edges of *E*(*S*_*u*_) are in both *S* and *S*^′^.

Therefore, *E*(*S*)∖*E*(*S*_*u*_)=*E*(*S*)∖*E*(*S*^′^) and *E*(*S*^′^)∖*E*(*S*_*u*_)=*E*(*S*^′^) ∖ *E*(*S*).

### **Lemma 16**.

Let *S* be an AST of  and let *e*={*u*,*v*} be an edge of *S*. Let *S*^′^ be the tree obtained by splitting *e* at *u*. Let *Ψ*, *Ψ*^′^ be the cut functions of *S* and *S*^′^ respectively. Consider any edge *f*∈*E*(*S*^′^)∖*E*(*S*). There exists an edge *e*^′^∈*E*(*S*)∖*E*(*S*^′^) where *Ψ*^′^(*f*)⊆*Ψ*(*e*^′^). Furthermore, if *Ψ*(*e*^′^) is a minimal cut of G(P) then *Ψ*^′^(*f*)=*Ψ*(*e*^′^) and *Ψ*^′^(*f*) is a minimal cut of G(P).

### *Proof*.

Let *f*={*x*,*y*} and let *x* be the vertex of *f* where *L*_*x*_⊆*L*_*v*_. Let *S*_*p*_ be the minimal subtree of *S* connecting the labels in *L*_*x*_. Let *p* be the vertex of *S*_*p*_ closest to *u* in *S*. Let *q* be the vertex adjacent to *p* in the path from *p* to *u*. Let *e*^′^={*p*,*q*}. Note that, *L*_*x*_⊆*L*_*p*_. Since *L*_*x*_⊆*L*_*v*_, *e*^′^ is an edge of *E*(*S*)∖*E*(*S*^′^). Consider any tree *T* that has an edge *f*_1_ in *Ψ*^′^(*f*). We show that $ℒ(T)\cap L_{x}=ℒ(T)\cap L_{p}$. It then follows that *f*_1_∈*Ψ*(*e*^′^) and thus, *Ψ*^′^(*f*)⊆*Ψ*(*e*^′^).

Since *L*_*x*_⊆*L*_*p*_, $\left(L_{x}\cap ℒ(T))\subseteq (ℒ(T)\cap L_{p}\right)$. By Observation 2(ii), all the labels in $ℒ(T)\cap L_{v}$ are in the same connected component of G(P)−Ψ(e). Thus, all the labels in $L_{x}\cup (L_{p}\cap ℒ(T))$ are in the same connected of G(P)−Ψ(e). If $\left(L_{p}\cap ℒ(T))⫅̸(L_{x}\cap ℒ(T)\right)$, then $S_{\left|(L_{x}\cup (L_{p}\cap ℒ(T)\right)}$ and $S_{\left|(L_{x}\cup (L_{p}\cap ℒ(T)\right)}^{\prime }$ are not isomorphic, contradicting Observation 3. Thus, $\left(L_{p}\cap ℒ(T))\subseteq (L_{x}\cap ℒ(T)\right)$

Assume that *Ψ*(*e*^′^) is a minimal cut of G(P). Then, all the labels in *L*_*p*_ are in the same connected component of $G(P)-\Psi (e^{\prime })$. By Observation 3, *L*_*p*_=*L*_*x*_. Thus, *Ψ*^′^(*f*) is also a minimal cut of G(P).

### **Lemma 17**.

Let *S* be an AST of  and *Ψ* be the cut function of *S*. Let *E*_0_ be the set of all edges *e* of *S* such that *Ψ*(*e*) is not a minimal cut of G(P). Choose any edge *e*^∗^={*u*,*v*}∈*E*_0_ such that $\left|\Psi (e^{*})|=\underset{e\in E_{0}}{\max}|\Psi (e)\right|$. Let *S*^′^ be the tree obtained from *S* by splitting *e*^∗^ at *u* and let *Ψ*^′^ be the cut function of *S*^′^. We have the following. 

1. For any edge *f*∈*E*(*S*^′^), if |*Ψ*^′^(*f*)|>|*Ψ*(*e*^∗^)| then *Ψ*^′^(*f*) is a minimal cut of G(P).

2. Let *P* be the set of all edges *x* in *S* such that |*Ψ*(*e*^∗^)|=|*Ψ*(*x*)| and *Ψ*(*x*) is not a minimal cut. Let *P*^′^ be the set of all edges *x* in *S*^′^ such that |*Ψ*(*e*^∗^)|=|*Ψ*(*x*)| and *Ψ*^′^(*x*) is not a minimal cut. Then, |*P*^′^|<|*P*|.

### *Proof*.

*(i)* Consider any edge *f*∈*E*(*S*^′^) where |*Ψ*^′^(*f*)|>|*Ψ*(*e*^∗^)|. If *f*∈*E*(*S*)∩*E*(*S*^′^), then *Ψ*(*f*)=*Ψ*^′^(*f*). Since |*Ψ*(*f*)|>|*Ψ*(*e*^∗^)|, by assumption *Ψ*(*f*) is a minimal cut of G(P). Thus, *Ψ*^′^(*f*) is also a minimal cut of G(P). Assume that *f*∈*E*(*S*^′^)∖*E*(*S*). By Lemma 16, there exists an edge *e*^′^∈*E*(*S*) where *Ψ*^′^(*f*)⊆*Ψ*(*e*^′^). Since |*Ψ*^′^(*f*)|>|*Ψ*(*e*^∗^)|, |*Ψ*(*e*^′^)|>|*Ψ*(*e*^∗^)|. Thus, by assumption *Ψ*(*e*^′^) is a minimal cut of G(P). From Lemma 16, it follows that *Ψ*(*e*^′^)=*Ψ*^′^(*f*) and *Ψ*^′^(*f*) is a minimal cut of G(P).

*(ii)* Let *Q*=*P*∩(*E*(*S*)∖*E*(*S*^′^)) and *Q*^′^=*P*^′^∩(*E*(*S*^′^)∖*E*(*S*)). It suffices to show that |*Q*^′^|<|*Q*|. Consider any edge *f*∈*Q*^′^. By Lemma 16, there exists an edge *e*^′^∈*E*(*S*)∖*E*(*S*^′^) where *Ψ*^′^(*f*)⊆*Ψ*(*e*^′^). Thus, |*Ψ*(*e*^′^)|≥|*Ψ*^′^(*f*)|. If |*Ψ*(*e*^′^)|>|*Ψ*^′^(*f*)|, then by assumption *Ψ*(*e*^′^) is a minimal cut and thus by Lemma 16 |*Ψ*(*e*^′^)|=|*Ψ*^′^(*f*)|, a contradiction.

Thus, *Ψ*(*e*^′^)=*Ψ*^′^(*f*). Also, since *Ψ*^′^(*f*) is not a minimal cut, by Lemma 16, neither is *Ψ*(*e*^′^). If *e*^′^=*e*^∗^, then all vertices of *V*_*v*_ are in the same connected component of G(P)−Ψ(e), contradicting the assumption that it is possible to split *e*^∗^ at *u*. Thus, *e*^′^≠*e*^∗^. Hence, we can conclude that for every edge *f*∈*Q*^′^, there exists an edge *e*^′^∈(*Q*∖{*e*^∗^}), where *Ψ*^′^(*f*)=*Ψ*(*e*^′^).

Let *f*_1_ and *f*_2_ be any two distinct edges in *Q*^′^. Let *e*_1_ and *e*_2_ be the edges of *Q*∖{*e*^∗^} where *Ψ*^′^(*f*_1_)=*Ψ*(*e*_1_) and *Ψ*^′^(*f*_2_)=*Ψ*(*e*_2_). If *e*_1_=*e*_2_, then *Ψ*^′^(*f*_1_)=*Ψ*^′^(*f*_2_), contradicting Observation 2(i). Thus, *e*_1_≠*e*_2_. Since *e*∈*Q* and *e*∉*Q*^′^, it follows that |*Q*^′^|≤|*Q*|−1, and thus |*Q*^′^|<|*Q*|.

### *Proof of Lemma 14*.

Let *S* be an AST of  and *Ψ* be the cut function of *S*. Do the following while *S* contains an edge *e* such that *Ψ*(*f*) is not a minimal cut of G(P): Pick an edge *e*^∗^ satisfying the conditions of Lemma 17, and apply a split operation at *e*^∗^; let *S*^′^ be the resulting tree. By Lemma 15, *S*^′^ is also an AST of . Let *Ψ*^′^ be the cut function of *S*^′^. Set *S* to *S*^′^ and *Ψ* to *Ψ*^′^

We only need to prove that the total number of iterations, *s*, is finite. An AST of  has at most 2|ℒ(P)| vertices. Also, |*Ψ*(*e*)|≥1 for any edge *e* of *S*. It thus follows from Lemma 17 that *s* is finite.

### *Proof of Lemma 12*

(⇐) Assume that  has an AST. Then, by Lemma 14,  has an AST *S* whose cut function *Ψ* has the property that, for every edge *e*∈*E*(*S*), *Ψ*(*e*) is a minimal cut of G(P). Let  be the set of all *Ψ*(*e*) such that *e* is an internal edge of *S*. Then,  is a set of minimal cuts of G(P). Further, by definition of *Ψ*, for every F∈F and for every T∈P, *F* contains at most one edge of *T*. Thus every cut in  is legal. We now prove that  is a complete set of pairwise parallel nice minimal cuts of G(P).

We first argue that every cut in  is nice. Consider any F∈F. Let *e*={*u*,*v*} be the internal edge of *S* where *Ψ*(*e*)=*F*. Let *T* be an input tree that has an internal edge *f* in *Ψ*(*e*). Since *e* is an internal edge at least one such input tree exists; otherwise *Ψ*(*e*) is not a minimal cut. Now, by definition, *f* is the only edge of *T* in *Ψ*(*e*), so, by Lemma 7, each of the two connected components of G(P)−Ψ(e) has at least one non-internal edge of *T*. Hence, *F* is a nice minimal cut of G(P).

To prove that the cuts in  are pairwise parallel, we argue that for any two distinct internal edges *e*_1_ and *e*_2_ of *S*, *Ψ*(*e*_1_) and *Ψ*(*e*_2_) are parallel. There exist vertices *x*∈*e*_1_ and *y*∈*e*_2_ where *L*_*x*_⊆*L*_*y*_. For every edge *f*∈*Ψ*(*e*_1_), we show that *f*∈*Ψ*(*e*_2_) or *f*⊆*V*_*y*_. It then follows that *Ψ*(*e*_1_) and *Ψ*(*e*_2_) are parallel. Let *f* be an edge of input tree *T*. Then there exists *z*∈*f* where *L*_*z*_⊆*L*_*x*_. Thus, *L*_*z*_⊆*L*_*y*_ and *z*∈*V*_*y*_. By Lemma 13, all the vertices of *V*_*y*_ are in the same connected component of $G(P)-\Psi (e_{2})$. Thus, *f*∈*Ψ*(*e*_2_) or *f*⊆*V*_*y*_.

Lastly, we show that  is complete. Consider any internal edge *f*={*p*,*q*} of some input tree *T*. Since *S* is an AST of , there exists an edge *e*={*u*,*v*} where, up to relabeling of sets, *L*_*p*_⊆*L*_*u*_ and *L*_*q*_⊆*L*_*v*_. Thus, *e* is an agreement edge of *S* corresponding to *f*, so *f*∈*Ψ*(*e*). Since *f* is an internal edge, *e* is also an internal edge of *S* and thus Ψ(e)∈F. Hence, for every internal edge *f* of an input tree there is a cut F∈F where *f*∈*F*. Thus, *§* is complete.

(⇒) Assume that there exists a complete set  of pairwise parallel nice minimal cuts of G(P) where, for every F∈F and every T∈P, *F* contains at most one edge of *T*. By Theorem 18, Σ(F) is compatible and, by Theorem 1, there exists an unrooted tree *S* where Σ(F)=Σ(S). We prove that *S* is an AST of  by showing that $\Sigma (S_{\left|ℒ(T\right)})=\Sigma (T)$ for every input tree T∈P.

Consider an input tree *T* of . Let *X*_1_|*X*_2_ be the non-trivial split of *T* corresponding to edge *f*∈*E*(*T*). Since  is complete, there exists a cut F∈F where F∈F. If *σ*(*F*)=*Y*_1_|*Y*_2_, by Lemma 7, up to relabeling of sets, *X*_*i*_⊆*Y*_*i*_ for every *i*∈{1,2}. Since *σ*(*F*) is a split of *S*, this implies that $\Sigma (T)\subseteq \Sigma (S_{\left|ℒ(T\right)})$.

Consider any non-trivial split *P*_1_|*P*_2_ of Σ(*S*) where $P_{i}\cap ℒ(T)\neq \emptyset$ for each *i*∈{1,2}. Let $Q_{i}=P_{i}\cap ℒ(T)$ for each *i*∈{1,2}. Since Σ(S)=Σ(F), there exists a cut F∈F where *σ*(*F*)=*P*_1_|*P*_2_. Since *P*_1_ and *P*_2_ are in different connected components of G(P)−F, *Q*_1_ and *Q*_2_ are also in different connected components of G(P)−F. Thus, *F* contains an edge *f*^′^ of *T*. Since *F* does not contain any other edge of *T*, *σ*(*f*^′^)=*Q*_1_|*Q*_2_. Thus, $\Sigma (S_{\left|ℒ(T\right)})\subseteq \Sigma (T)$.

## Relationship to legal triangulations

Taken together, Theorems 3 and 6 say that G(P) has a complete set of pairwise parallel legal minimal cuts if and only if it has a legal triangulation. The connection between legal triangulations and complete sets of pairwise parallel legal minimal cuts is through the existence (or nonexistence) of a compatible tree. Here we make the connection explicit, showing how, from a set of pairwise parallel legal minimal cuts, one can construct a legal triangulation of G(P) without going through a compatible tree. We leave the other direction —going from a triangulation to a set of cuts— to the reader.

Let  be a complete set of pairwise parallel legal minimal cuts of G(P). We assume that the elements of  are ordered in some arbitrary, but fixed, manner, and that no proper subset of  is also complete. For each F∈F, we build a pair (*X*_*F*_,*Y*_*F*_) where *X*_*F*_ and *Y*_*F*_ are vertex separators of G(P), and *X*_*F*_,*Y*_*F*_⊆{*u*:*u* is the endpoint of some edge in*F*}. The collection of pairs $\left\{(X_{F},Y_{F}):F\in F\right\}$ is not unique, as it depends on the order in which  is arranged. We say that a cut F∈F*differentiates* an internal edge *e*={*x*,*y*} if *x*∈*X*_*F*_ and *y*∈*Y*_*F*_.

For each F∈F, let *F*_*i*_=*E*(*T*_*i*_)∩*F* for each *i*∈[*k*], and let $\hat{F}\subseteq F$ denote the set of all edges *e* such that *e*∈*F*_*i*_ for some *i*∈[*k*] with |*F*_*i*_|=1. Note that if |*F*_*i*_|>1, all edges in *F*_*i*_ must share a common endpoint. Let *A*_*F*_ and *B*_*F*_ denote the two connected components of G(P)−F.

For each cut *F* in , we build (*X*_*F*_,*Y*_*F*_) as follows. 

1. For each internal edge $e\in \hat{F}$: 

(a) If no cut preceding *F* differentiates *e*, add *e*∩*V*(*A*_*F*_) to *X*_*F*_ and *e*∩*V*(*B*_*F*_) to *Y*_*F*_.

(b) Otherwise, suppose cut I∈F, which precedes *F*, differentiates *e*. Let *Q* be the connected component of G(P)−I where *E*(*Q*)∩*F*≠∅. (Note that *Q* is unique, since *I* and *F* are parallel.) Let *v* be the unique endpoint of *e* in *Q*. Add *v* to *X*_*F*_ and *Y*_*F*_.

2. For each non-internal edge $e\in \hat{F}$, add the non-leaf endpoint of *e* to both *X*_*F*_ and *Y*_*F*_.

3. For each *i*∈[*k*] such that |*F*_*i*_|>1, add the common endpoint of the edges of *F*_*i*_ to both *X*_*F*_ and *Y*_*F*_.

By construction and the properties of , every edge internal edge of G(P) is differentiated by some cut F∈F. Further, the sets *X*_*F*_ and *Y*_*F*_ have the form *X*_*F*_={*x*_1_,…,*x*_*m*_,*z*_1_,…,*z*_*p*_} and *Y*_*F*_={*y*_1_,…,*y*_*m*_,*z*_1_,…,*z*_*p*_}, where *m*>0, *p*≥0, and for every *i*∈[*m*], {*x*_*i*_,*y*_*i*_} is an internal edge of G(P) that is differentiated by *F*. Let

$$O_{F}=\left\{\left\{x_{1},\ldots ,x_{j},y_{j},\ldots ,y_{m},z_{1},\ldots ,z_{p}\}:j\in [m\right]\right\}.$$

We now state how to go from a complete set of pairwise parallel legal cuts to a legal triangulation. As in Section ‘Preliminaries’, given a graph *G* and a collection Δ of subsets of *V*(*G*), *G*_Δ_ denotes the graph derived from *G* by making the set of vertices of *X* a clique for every *X*∈Δ.

### **Theorem 11**.

Let Δ be the collection of subsets of V(G(P)) given by

(1) $$\Delta =\{N_{G(P)}(\ell ):\ell \;\text{is a leaf in}\;G\;(P)\}\cup \underset{F\in F}{⋃}(\{X_{F},Y_{F}\}\cup O_{F}).$$

Then, $G{\left(P\right)}_{\Delta }$ is a legal triangulation of G(P).

The proof of Theorem 11 relies on a series of auxiliary lemmas, for which we introduce some new notation. For each F∈F, *F*_∪_ denotes *X*_*F*_∪*Y*_*F*_ and *F*_∩_ denotes *X*_*F*_∩*Y*_*F*_. Also, we abbreviate $G{\left(P\right)}_{\Delta }$ to *G*_Δ_, where Δ is the set defined in Equation (1)

### **Lemma 18**.

Let *F* and *I* be two distinct cuts of , and let *x* be a vertex of *F*_∪_. Suppose *x* lies in the connected component of G(P)−I that does not contain edges of *F*. Then, *x*∈*I*_∩_.

### *Proof*.

Let *E*_*F*,*x*_ be the set of all edges of *F* that contain *x* and let *E*_*I*,*x*_ be the set of all edges of *I* that contain *x*. We must have *E*_*F*,*x*_⊆*E*_*I*,*x*_⊆*I*. If |*E*_*I*,*x*_|>1, then *x*∈*I*_∩_. Thus, assume that |*E*_*I*,*x*_|=1. Let *E*_*I*,*x*_={*e*}, where *e*={*x*,*y*}. Since *E*_*F*,*x*_⊆*E*_*I*,*x*_ and |*E*_*F*,*x*_|≥1, *E*_*F*,*x*_={*e*}. We can assume that *y* is not a leaf (since, otherwise, *x*∈*I*_∩_). Let *E*_*I*,*y*_ be the set of edges of *I* with *y* as an endpoint. Vertex *y* lies in the component of *G*−*F* that does not contain *I*. Thus, every edge in *E*_*I*,*y*_ is also present in *F*. If |*E*_*I*,*y*_|>1, then there is more than one edge in *F* with *y* as an endpoint and by construction, *x*∉*F*_∪_. Hence, |*E*_*I*,*y*_|=1, and so *E*_*I*,*y*_={*e*}.

Let *J* be the cut that differentiates *e*. If *F*=*J* then by construction, *x*∈*I*_∩_. Thus, assume that *F*≠*J*. If *J* is in the same connected component of G(P)−F as *I*, then, by construction *x*∉*F*_∪_, which is a contradiction. Thus, *J* is in the connected component of G(P)−F that does not contain *I* and, by construction, *x*∈*I*_∩_.

### **Lemma 19**.

Let F∈F. For every edge {*u*,*v*} in *G*_Δ_, (i) if *u*∈*V*(*A*_*F*_)∖*F*_∩_, then *v*∉*V*(*B*_*F*_)∖*Y*_*F*_, and (ii) if *u*∈*V*(*B*_*F*_)∖*F*_∩_, then *v*∉*V*(*A*_*F*_)∖*X*_*F*_.

### *Proof*.

Without loss of generality, we consider only the case where *u*∈*V*(*A*_*F*_)∖*F*_∩_. Suppose that *v*∈*V*(*B*_*F*_)∖*Y*_*F*_. If e∈E(G(P)), then *e*∈*F* and hence, by construction, at least one of *u* and *v* is in *F*_∪_. But *v*∉*Y*_*F*_, so *u*∈*F*_∩_, a contradiction.

Thus, *e* must be a fill-in edge. Since *e* ⊈ *F*_∪_, there must be a cut I∈F, *I*≠*F*, such that *e*⊆*I*_∪_. If *E*(*A*_*F*_)∩*I*≠∅, then by Lemma 18, *v*∈*F*_∩_, a contradiction. Thus, assume that *E*(*B*_*F*_)∩*I*≠∅. Then, by Lemma 18, *u*∈*F*_∩_, another contradiction

A clique of *G*_Δ_ is *illegal* if it contains a fill-in edge with a leaf as an endpoint or it contains an internal edge along with any another edge of G(P). An illegal clique violates one of the legal triangulation conditions (LT1) or (LT2) stated in Section ‘Display graphs and edge label intersection graphs’.

### **Lemma 20**.

Let *F* be a cut of  and let *H* be the subgraph of *G*_Δ_ induced by vertices of *F*_∪_. Then, *H* is triangulated and contains no illegal clique.

### *Proof*.

Let *X*_*F*_={*x*_1_,…,*x*_*m*_,*z*_1_,…,*z*_*p*_} and *Y*_*F*_={*y*_1_,…,*y*_*m*_,*z*_1_,…,*z*_*p*_}, where for every *i*∈[*m*], *x*_*i*_∈*V*(*A*_*F*_), *y*_*i*_∈*V*(*B*_*F*_) and {*x*_*i*_,*y*_*i*_} is an internal edge of G(P). Note that *F*_∩_={*z*_1_,…,*z*_*p*_}.

**Claim.***For everyi*,*j*∈[*m*] where *i*>*j*, *e*={*x*_*i*_,*y*_*j*_}∉*E*(*H).*

### *Proof*.

Assume that *e*∈*E*(*H*). By construction of (*X*_*F*_,*Y*_*F*_), *e* is a fill-in edge. Since no set in *O*_*F*_ contains both *x*_*i*_ and *y*_*j*_, there is a cut I∈F where *e*⊆*I*_∪_. Since *F* and *I* are parallel, only one of the two sets *I*∩*E*(*A*_*F*_) or *I*∩*E*(*B*_*F*_) is non-empty. Assume that *I*∩*E*(*A*_*F*_)≠∅. Then by Lemma 18, *y*_*j*_∈*F*_∩_, a contradiction. Similarly, if *I*∩*E*(*B*_*F*_)≠∅, then by Lemma 18, *x*_*i*_∈*F*_∩_, a contradiction.

Let *C* be a chordless cycle of length at least four in *H*. Since *X*_*F*_ and *Y*_*F*_ are cliques in *G*_Δ_, if *C* contains more than two vertices from one of *X*_*F*_ or *Y*_*F*_, then *C* must contain a chord. Hence, *C* has exactly four vertices, with exactly two vertices each from *X*_*F*_ and *Y*_*F*_. We will first show that *z*_*i*_∉*C* for any *i*∈*p*. Assume that *z*_*i*_∈*C* for some *i*∈[*p*]. Then, of the remaining three vertices of *C*, at least two of them belong to one of *X*_*F*_ and *Y*_*F*_. Let *a*, *b* be those two vertices. Without loss of generality assume that {*a*,*b*}⊆*X*_*F*_. Since, *F*_∩_⊆*X*_*F*_, vertices *z*_*i*_, *a*, *b* form a clique in *H*. Thus, *C* is not chordless, a contradiction.

Let *x*_*i*_,*x*_*j*_ be the vertices of *X*_*F*_ in *C* where 1≤*i*<*j*≤*m*. Similarly, let $y_{i^{\prime }},y_{j^{\prime }}$ be the vertices of *Y*_*F*_ in *C* where 1≤*i*^′^<*j*^′^≤*m*. Now, either *i*≤*i*^′^ or *i*>*i*^′^. If *i*≤*i*^′^, then {*x*_1_,…,*x*_*i*_,*y*_*i*_,…,*y*_*m*_,*z*_1_,…,*z*_*p*_}∈*O*_*F*_ and thus vertices $x_{i},y_{i^{\prime }},y_{j^{\prime }}$ form a clique. Hence, *C* is not chordless, a contradiction. If *i*>*i*^′^, then from the above claim neither of the edges $\left\{x_{i},y_{i^{\prime }}\right\}$ and $\left\{x_{j},y_{i^{\prime }}\right\}$ can exist. Thus, vertex $y_{i^{\prime }}$ cannot be in *C*, a contradiction. Hence, *H* does not contain a chordless cycle and is triangulated.

Assume that *H* contains an illegal clique *H*^′^; that is, *H*^′^ contains two internal edges *e* and *e*^′^. By construction, *F*_∪_ cannot contain a leaf. By the legality of *F* and the construction of *F*_∪_, edges *e* and *e*^′^ are from different input trees and both are differentiated by *F*. Let *e*={*x*_*i*_,*y*_*i*_} for some *i*∈[*m*] and let *e*^′^={*x*_*j*_,*y*_*j*_} for some *j*∈[*m*]. Without loss of generality, assume that *i*<*j*. By the above claim, there is no edge between *x*_*j*_ and *y*_*i*_ in *H*; thus, *H*^′^ is not a clique, a contradiction. ***□***

### **Lemma 21**.

*G*_Δ_ is chordal.

### *Proof*.

Assume the contrary. Let *C* be a chordless cycle of length at least four in *G*_Δ_. By construction, *C* cannot contain a leaf. There are two cases. *Case 1:There are vertices u*,*v*∈*V*(*C*) and a cut F∈F where *u*∈*X*_*F*_∖*F*_∩_ and *v*∈*Y*_*F*_∖*F*_∩_*.*

We have two subcases. *Case 2:There is no cut*F∈F with vertices *u*∈*X*_*F*_∖*F*_∩_ and *v*∈*Y*_*F*_∖*F*_∩_ such that *u*,*v*∈*V*(*C).* Thus, for every cut F∈F at most two vertices of *V*(*C*) are in *F*_∪_. Let *x*_1_,*x*_2_,*x*_3_,*x*_4_ be a path of length four in *C*. For every *i*∈{1,2,3}, let $F^{\left(i\right)}\in F$ be the cut where $\{x_{i},x_{i+1}\}\subseteq F_{\cup }^{\left(i\right)}$. We will first show that such cuts exist and are distinct.

(a) *Suppose C contains a vertexx*∈*F*_∩_*.* Then, there exists a path *u*,*x*,*v* in *C*. Because *C* is a cycle, there must exist an edge between a vertex *u*^′^∈*V*(*A*_*F*_)∖*x* and *v*^′^∈*V*(*B*_*F*_)∖*x*. Since *C* is chordless, *u*^′^∉*F*_∩_ and *v*^′^∉*F*_∩_. Thus, *u*^′^∈*V*(*A*_*F*_)∖*F*_∩_ and *v*^′^∈*V*(*B*_*F*_)∖*F*_∩_. By Lemma 19, if *u*^′^∈*V*(*A*_*F*_)∖*X*_*F*_ then there is no edge between *u*^′^ and *v*^′^. Thus, *u*^′^∈*X*_*F*_∖*F*_∩_. Similarly, *v*^′^∈*Y*_*F*_∖*F*_∩_. If *u*≠*u*^′^ or *v*≠*v*^′^, *C* cannot be chordless. Thus, *u*=*u*^′^ and *v*=*v*^′^ and *C* has length three, a contradiction

(b) *Suppose C does not contain a vertex ofF*_∩_*.* Since *u*∈*V*(*A*_*F*_)∖*F*_∩_, *v*∈*V*(*B*_*F*_)∖*F*_∩_ and *F* is a cut, there must exist two edges *e*_1_={*x*_1_,*y*_1_} and *e*_2_={*x*_2_,*y*_2_} in *C* where {*x*_1_,*x*_2_}⊆*V*(*A*_*F*_)∖*F*_∩_ and {*y*_1_,*y*_2_}⊆*V*(*B*_*F*_)∖*F*_∩_. If *x*_1_∈*V*(*A*_*F*_)∖*X*_*F*_, then by Lemma 19 there cannot exist an edge between *x*_1_ and *y*_1_. Thus, *x*_1_∈*X*_*F*_∖*F*_∩_. Similarly, *x*_2_∈*X*_*F*_∖*F*_∩_ and {*y*_1_,*y*_2_}⊆*Y*_*F*_∖*F*_∩_. Since *X*_*F*_ and *Y*_*F*_ are cliques in *G*_Δ_, there exist edges {*x*_1_,*x*_2_} and {*y*_1_,*y*_2_}. Thus, there cannot exist any other vertex in *C* and hence *V*(*C*)⊆*F*_∪_. But, by Lemma 20 subgraph of *G*_Δ_ induced by vertices of *F*_∪_ is triangulated. Thus, *C* is not chordless, a contradiction

Recall that every vertex in *C* is internal. Also, *C* does not contain any edge *e*={*x*,*y*} from *G*; otherwise, there would be a cut *F*^′^ that differentiates *e*, contradicting the assumption for case 2. Since every edge in *C* is in *G*_Δ_, it must be the case that for every edge *e* in *C* there exists a cut *F* where *e*⊆*F*_∪_. Also, at most two vertices of *C* are in *F*_∪_. Thus the cuts *F*^(1)^, *F*^(2)^ and *F*^(3)^ are distinct.

To simplify notation, for each *i*∈{1,2,3} let $A_{i}=A_{F^{\left(i\right)}}$ and $B_{i}=B_{F^{\left(i\right)}}$. Without loss of generality, assume that *E*(*A*_1_)∩*F*^(2)^≠∅ and *E*(*B*_2_)∩*F*^(1)^≠∅. There are three possibilities. 

(a) *SupposeF*^(3)^∩*E*(*A*_2_)≠∅*.* If *x*_1_∈*A*_2_, then by Lemma 18, $x_{1}\in F_{\cap }^{\left(2\right)}$ and *C* is not chordless, a contradiction. Thus, *x*_1_∈*B*_2_. Similarly, if *x*_4_∈*B*_2_, by Lemma 18, $x_{4}\in F_{\cap }^{\left(2\right)}$ and *C* is not chordless, a contradiction. Thus, *x*_4_∈*A*_2_. Since *C* is a cycle, *F*^(2)^ is a minimal cut and $\{F_{\cup }^{\left(2\right)}\setminus \{x_{2},x_{3}\}\}\cap V(C)=\emptyset$, there exists an edge {*v*_1_,*v*_2_} in *C* where $v_{1}\in V(A_{2})\setminus F_{\cup }^{\left(2\right)}$ and $v_{2}\in V(B_{2})\setminus F_{\cup }^{\left(2\right)}$. But, by Lemma 19, such an edge cannot exist.

(b) *SupposeF*^(3)^∩*E*(*A*_1_)≠∅ and *F*^(3)^∩*E*(*B*_2_)≠∅*.* Without loss of generality, assume that *A*_3_, *B*_3_ contain *F*^(2)^ and *F*^(1)^ respectively. Assume that *x*_2_∈*A*_3_. Since $x_{2}\in F_{\cup }^{\left(1\right)}$, by Lemma 18, $x_{2}\in F_{\cap }^{\left(3\right)}$. Then, there exists an edge {*x*_2_,*x*_4_} and *C* is not chordless, a contradiction. Thus, *x*_2_∈*B*_3_. But $x_{2}\in F_{\cup }^{\left(2\right)}$ and thus, by Lemma 18, $x_{2}\in F_{\cap }^{\left(3\right)}$. Hence, there exists a chord {*x*_2_,*x*_4_} and *C* is not chordless, again a contradiction.

(c) *SupposeF*^(3)^∩*E*(*B*_1_)≠∅*.* Renaming vertices *x*_1_, *x*_2_, *x*_3_ and *x*_4_ as, *x*_4_, *x*_3_, *x*_2_ and *x*_1_, respectively, brings us back to subcase 2(b).

Thus, *G*_Δ_ does not contain a chordless cycle of length four or greater; hence, *G*_Δ_ is chordal.

### *Proof of Theorem 11*.

Lemma 21 states that *G*_Δ_ is triangulated. We now prove that *G*_Δ_ is a legal triangulation; i.e., that it satisfies conditions (LT1) and (LT2) of Section ‘Display graphs and edge label intersection graphs’

Condition (LT2) holds for *G*_Δ_, because our construction adds no fill-in edge incident on a leaf. Now suppose that *G*_Δ_ violates (LT1); i.e., *G*_Δ_ has a clique *H* with two internal edges *e*={*x*_1_,*y*_1_} and *e*^′^={*x*_2_,*y*_2_}. Let *F* be the cut that differentiates *e*. Assume that *x*_1_∈*V*(*A*_*F*_) and *y*_1_∈*V*(*B*_*F*_). By Lemma 20, *F*_∪_ does not contain both endpoints of *e*^′^. Without loss of generality, assume that *x*_2_∉*F*_∪_ and *x*_2_∈*A*. Since *x*_2_∉*F*_∪_ and *y*_1_∉*F*_∩_, by Lemma 19, there is no edge between *x*_2_ and *y*_1_ in *G*_Δ_. Thus, *H* is not a clique of *G*_Δ_, a contradiction. Hence, *G*_Δ_ satisfies (LT1) and is therefore a legal triangulation of G(P).

## Conclusion

We have shown that the characterization of tree compatibility in terms of restricted triangulations of the edge label intersection graph transforms into a characterization in terms of minimal cuts in the display graph. These two characterizations are closely related to the legal triangulation characterization of [8]. We also derived characterizations of the agreement supertree problem in terms of minimal cuts and minimal separators of the display and edge label intersection graphs respectively.

It remains to be seen whether any of our characterizations can lead to explicit fixed-parameter algorithms for the tree compatibility and agreement supertree problems when parametrized by the number of trees. Indeed, as of yet, the fixed-parameter tractability of agreement remains open.

We close with some remarks on characterizations of two problems related to compatibility. A profile *defines* a tree *S* if *S* is the only compatible supertree for . *identifies* a tree *S* if *S* is a compatible supertree for  and every other compatible supertree for  displays *S*. Grunewald et al. [16] use quartet graphs to characterize when a profile consisting of quartet trees defines or identifies a tree. An interesting question is whether similar characterizations can be derived for arbitrary profiles using display graphs or edge label intersection graphs. Along these lines, we note a connection between complete sets of cuts and the question of whether a profile defines a tree, which was pointed out by one of the reviewers. To explain it, we need some definitions ([10], p. 131). Let *T* be a tree and let *q*=*xy*|*wz* be a quartet tree displayed by *T*. Quartet tree *qdistinguishes* an interior edge *e* of *T* if *e* is the only interior edge such that {*x*,*y*} and {*w*,*z*} are in different connected components of *T*−*e*. Now, let *S* and *T* be two trees such that *S* displays *T*. An interior edge *e* of *Tdistinguishes* an interior edge *f* of *S* if there exists a quartet *q* such that *e* and *f* are both distinguished by *q*. Suppose  is a profile in which there is at least one taxon in common among all input trees. Then,  defines a tree *S* if and only if  is compatible and every interior edge of *S* is distinguished by an interior edge of at least one tree in  ([10], p. 133). Now, recall that if  is a complete set of cuts of G(P), then, for every tree $T_{i}\in P$ and every internal edge *e* of *T*_*i*_, there is some cut F∈F in which *e* is the only edge of *T*_*i*_. Thus, if  is compatible, *e* must be a distinguishing edge for some internal edge of a supertree for . This observation could lead to a cut-based characterization of definability analogous to known triangulation-based characterizations (see [10], p. 79).

## Competing interests

The authors declare that they have no competing interests.

## Authors’ contributions

SV stated and proved the main results of the paper and wrote most of the first draft. DFB proposed the research topic to SV, supervised the research, contributed to the first draft, and was in charge of the final draft. Both authors read and approved the final manuscript.
